# Childhood exposure to particulate matter and nitrogen oxides and associations with mental health disorders in early adulthood: testing mediation by cognition in a UK longitudinal cohort study

**DOI:** 10.1136/bmjment-2025-301864

**Published:** 2025-09-30

**Authors:** Thomas Canning, Louise Arseneault, Rachel M Latham, Joanne B Newbury, Aaron Reuben, Ioannis Bakolis, Helen L Fisher

**Affiliations:** 1 Department of Biostatistics and Health Informatics, Institute of Psychiatry, Psychology & Neuroscience, King’s College London, London, UK; 2 Social, Genetic & Developmental Psychiatry Centre, Institute of Psychiatry, Psychology & Neuroscience, King’s College London, London, UK; 3 Centre for Mental Health Policy and Evaluation, Health Service and Population Research Department, Institute of Psychiatry, Psychology & Neuroscience, King’s College London, London, UK; 4 ESRC Centre for Society and Mental Health, King’s College London, London, UK; 5 Population Health Sciences, Bristol Medical School, University of Bristol, Bristol, UK; 6 Department of Psychology, University of Virginia, Charlottesville, Virginia, USA

**Keywords:** Mental Health, Depressive Disorder, Anxiety Disorders, Attention Deficit and Disruptive Behavior Disorders

## Abstract

**Background:**

Little is known about the mechanisms underlying associations between air pollution exposure in childhood and mental health disorders in adulthood.

**Objective:**

To examine the prospective associations between age-10 air pollution exposure and age-18 mental health disorders and to test potential mediation by impaired cognition at age 12.

**Methods:**

We used longitudinal observations of 1969 members of the Environmental Risk Study who were born across England and Wales in 1994–1995. Exposure to nitrogen dioxide, nitrogen oxides (NO_x_) and particulate matter (PM_2.5_ and PM_10_) was modelled for residential addresses at age 10. Past-year prevalence of anxiety, depression, conduct disorder and attention-deficit/hyperactivity disorder was assessed by interview at age 18. Overall cognition (full-scale IQ) and specific domains (fluid ability, crystallised ability and working memory) were assessed at age 12. We employed binary logistic regression to examine pollution–disorder associations and generalised structural equation modelling to examine mediation via impaired cognition.

**Findings:**

Higher exposure to NO_x_ was associated with greater odds of depression after covariate adjustment (OR=1.25, 95% CI 1.01 to 1.55). No robust associations were evident for the other pollutants or outcomes. Overall cognition (indirect effect (IE): OR=1.00, 95% CI 0.99 to 1.01) and crystallised ability (IE: OR=1.00, 95% CI 0.99 to 1.01) did not mediate the association between NO_x_ and depression.

**Conclusions:**

We found no evidence that impaired cognition mediated associations between childhood residential exposure to NO_x_ and depression in early adulthood.

**Clinical implications:**

Policies to reduce childhood exposure to NO_x_ may help reduce depression in early adulthood. Future research should examine alternative mechanisms.

**WHAT IS ALREADY KNOWN ON THIS TOPIC**

- There is mixed evidence that air pollution exposure in early life may elevate mental health disorder risk in young adults.
- Evidence on potential mechanisms underlying these associations is extremely limited.
- Cognitive deficits reflect one proposed mediating mechanism that has yet to be evaluated.

**WHAT THIS STUDY ADDS**

- This study extends existing evidence of associations of higher exposure to nitrogen oxides (NO_x_) in childhood and increased odds of depression in early adulthood.
- Findings also suggest that overall cognitive functioning and crystallised ability in early adolescence are not mediators of this association.

**HOW THIS STUDY MIGHT AFFECT RESEARCH, PRACTICE OR POLICY**

- This study indicates that, if associations are replicated further, action to reduce NO_x_ exposure in childhood and adherence to WHO recommendations may mean fewer young adults develop depression.
- Interventions on the air pollution–depression pathway may need to target other cognitive and non-cognitive factors.
- Further research is necessary to determine which putative mediating mechanisms are at play in air pollution–mental health disorder associations.

## Background

 Addressing mental ill health is a key global health priority as it is one of the leading causes of disability worldwide.^1^ Between half and three-quarters of people with mental health problems experience their onset by 18 years of age.^2^ Outdoor air pollution is the largest environmental health risk and one of the most prominent risk factors for ill health globally.^3^ Exposure to various air pollutants has been associated with a range of physical health problems, including cardiovascular and respiratory problems.^3^ There is also a growing evidence base for associations with mental health disorders.^4^ However, there remains limited consistent evidence across the different types of pollutants and mental health-related conditions. Previous studies in young people have also tended to use samples limited to a single city^5 6^ or focused on continuous symptom dimensions, which may present challenges for interpretation on a population scale.^7 8^ Therefore, further investigation is required of the associations between exposure to air pollutants in childhood and the development of mental health disorders by early adulthood in studies covering wider populations. Focusing on associations with mental health disorders may also help to facilitate seamless translation into policy and health system changes as services are often designed around such diagnostic categories.

Furthermore, it remains unclear as to what the mechanistic pathways are through which early exposure to air pollution may result in the subsequent development of mental health disorders.^9 10^ Understanding the mechanisms underlying these associations is important to help inform policies and interventions to mitigate the potentially adverse effects of air pollution on mental health.^9^ There are putative primary physical mechanisms for action. Particulate matter (PM) and gaseous pollutants (such as nitrogen oxides (NO_x_)) may trigger systemic inflammatory processes, or in the case of PM_2.5_ (PM smaller than 2.5 µm) through neurotoxic action after direct transportation to the brain.^11^ Secondary processes, including neurodegeneration, oxidative stress or disruption to cortical networks, may lead to functional changes in processing and therefore cognition in young people.^10 11^

Cognition has been associated with subsequent poorer general psychopathology, behavioural problems or anxiety^12^ and, in some cohorts, with depression.^13^ Cognition in general is of particular interest as it has a strong evidence base for causative associations with air pollution exposure.^14^ In older adult cohorts, composite cognitive measures have shown mediation of air pollution associations with mood disorders.^15^ In younger people, it is possible that they may be particularly at risk for cognitive impairment after exposure due to their developing brain (eg, immature blood–brain barrier) and general physiology (eg, a developing immune system or lung capacity). Deficiencies in cognitive processes in participants with prior higher air pollution exposure, such as fluid or crystallised ability and working memory, may then lead to increased risk for mental health disorders towards early adulthood.^16 17^ Identifying whether overall cognition, or specific domains, mediates air pollution–mental health disorder associations may therefore offer opportunities to plan remediation interventions or inform air pollution reduction policies.

### Objective

To examine prospective associations between air pollution exposure at age 10 and the presence of major depressive disorder (MDD), generalised anxiety disorder (GAD), attention-deficit/hyperactivity disorder (ADHD) and conduct disorder at age 18 by capitalising on data from a nationally representative longitudinal birth cohort study. Second, to test potential mediation of these associations by working memory, fluid and crystallised ability and overall cognitive functioning at age 12. We hypothesise that higher pollutant exposure will increase the odds of having any of these mental health disorders at age 18 and that this association will be partially mediated by poorer cognition at age 12.

## Methods

Participants were members of the Environmental Risk (E-Risk) Longitudinal Twin Study, which tracks the development of a nationally representative cohort of 2232 twins born in 1994–1995 across England and Wales and initially assessed at 5 years of age.^18^ Follow-up home visits were conducted when participants were age 7 (98% participation), 10 (96%), 12 (96%) and 18 (93%). At age 18, the E-Risk sample included 2066 participants, comprising 56.2% monozygotic twin pairs and 47.5% males. There were no differences between those who did and did not take part at age 18 in terms of age-5 socioeconomic status (SES) (p=0.65), age-5 IQ scores (p=0.33), or age-5 internalising or externalising behavioural problems (p=0.69 and p=0.68, respectively). E-Risk families are representative of UK households across the spectrum of neighbourhood socioeconomic conditions (online supplemental eFigure 1). A full description of the cohort is provided in online supplemental methods and a participant flowchart in online supplemental eFigure 2.

This paper followed the Strengthening the Reporting of Observational Studies in Epidemiology reporting guidelines (online supplemental eTable 1). The analysis plan was preregistered (https://sites.duke.edu/moffittcaspiprojects/files/2024/08/Canning_2024_Pollution-cognition-and-mental-disorders.pdf).

### Measures

#### Air pollution

Exposures were modelled by the coupled regional chemical transport model and street-scale dispersion model Community Multiscale Air Quality (CMAQ)-Urban at age 10 and age 18, as previously described.^19^ As we required a temporal sequence for mediation analysis, only age-10 air pollution exposure was used in this analysis. Included pollutants were nitrogen dioxide (NO_2_), nitrogen oxides (NO_x_), PM_2.5_ and PM smaller than 10 µm (PM_10_). Pollutants were modelled at a 20 m × 20 m resolution at household addresses for participants as an annual average at age 10 (2004). Further details, including model performance for all pollutants, are provided in online supplemental methods and eTable 2.

#### Mental health

At age 18, mental health disorders were assessed via private structured interviews using the Diagnostic Interview Schedule administered by trained interviewers as previously described.^20^ Past year symptoms were comprehensively assessed for GAD, MDD, ADHD and conduct disorder. These were then diagnosed using Diagnostic and Statistical Manual of Mental Disorders (DSM)—IV criteria (DSM-V for ADHD). Diagnostic cut-offs were based on the presence of symptom criteria (eg, for MDD at least five symptoms were required to be present, plus interference in daily functioning). Participants were then assigned a disorder status as binary variables (0=no, 1=yes) for each disorder. Around a fifth of the sample met criteria for MDD at age 18 (20.3%), with the other disorders being less prevalent (7.4% GAD, 8.1% ADHD, 15.2% conduct disorder). Further details are provided in online supplemental methods.

#### Cognition

Overall cognitive ability (full-scale IQ) was assessed at age 12 using the short form of the Wechsler Intelligence Scale for Children—IV.^21^ This was prorated to a score of 100 for the age group using the Information and Matrix Reasoning tasks, as described by Sattler and Dumont.^22^ We further examined three cognition subdomains, including crystallised ability (information task), fluid ability (matrix reasoning) and working memory (digit span task).

#### Covariates

Several covariates were included, with further details available in online supplemental methods. Assigned sex at birth was included as a covariate. Family SES was measured at age 5 through a standardised composite of parental income, occupation and education. These variables were highly correlated and loaded onto one latent factor, which was then categorised into tertiles, representing low, medium and high SES, to improve interpretability of the score. Neighbourhood deprivation was ascertained using a classification system based on a geodemographic discriminator using >400 census variables for Great Britain (CACI Information Services; http://www.caci.co.uk/), which was linked to home postcodes at ages 5, 7 and 10, and then averaged and rounded, across ages 5–10. The classifications were: ‘wealthy achiever’” (coded 1), ‘urban prosperity’ (2), ‘comfortably off’ (3), ‘moderate means’ (4) and ‘hard pressed’ (5) neighbourhoods. We also included an urbanicity measure, averaging and rounding urbanicity ratings (1=rural, 2=urban city/town, 3=major/minor conurbation) at ages 5, 7 and 10 into a single score. Family psychiatric history was assessed at age 12 through private interviews with mothers who reported on their history of mental health disorders and that of their biological mothers, fathers, sisters, brothers and the twin’s biological father. This was included as a proportion of family members with a psychiatric history (range 0–1). Finally, at age 18 years, participants were asked if they were ever a daily cigarette smoker, with a binary response given (0=no or 1=yes).

### Statistical analysis

All analyses were completed in STATA V.18.0 MP. We only included participants who had full exposure and outcome information. All pollutants were rescaled by the IQR of exposure for the analytic sample, to allow for comparison between pollutants that are on different scales of exposure. All analyses were adjusted to account for the non-independence of twin observations using the Huber-White variance estimator through the CLUSTER command.^23^

Covariate and mediator data missingness was low but was handled with multiple imputation through chained equations (online supplemental methods). We examined correlations between air pollutant exposures by calculating Pearson correlation coefficients.

#### Air pollution and mental health

We examined associations among NO_2_, NO_x_, PM_2.5_ and PM_10_ separately at age 10 with each diagnostic outcome of GAD, MDD, ADHD and conduct disorder at age 18 through logistic regression models. Analyses were first conducted without any covariates and then rerun including assigned sex at birth, family SES (assessed at age 5), neighbourhood deprivation (averaged across ages 5–10), urbanicity (averaged across ages 5–10), family psychiatric history (assessed at age 12) and tobacco smoking up to 18 years of age. All associations are reported with ORs and 95% CIs), representing the increased odds for a disorder per IQR increase in exposure.

#### Air pollution and overall cognition

We a priori planned to examine mediation only in exposure–outcome pairs that had significant associations in the prior analysis. We constructed directed acyclic graphs for the mediation analysis (online supplemental eFigure 3). We first assessed associations between exposure at age 10 and overall cognition at age 12 with the use of linear regression models. We then employed generalised structural equation modelling to test different pathways of the effect of exposure to each pollutant on each mental health disorder via overall cognition using the *gsem* command. We included covariates as shown in online supplemental eFigure 3. We calculated the total effect, the direct effect (DE) and indirect effect (IE) of exposure on mental health outcomes for exposure–outcome pairs that had significant main associations in fully adjusted models. The DE represents the effect of air pollution exposure on odds of mental health that is independent of the exposure’s effect through cognition (the IE). In online supplemental eFigure 3, the IE is represented by the product of the paths *ab*, the DE is equal to *c′* and the total effect (*c*) is equal to *a×b+c′*. We did not standardise coefficients as this would not meaningfully improve estimation bias.^24^ We used the CLUSTER command to recover the correct SEs for direct and IEs and to account for non-independence of twin observations. All associations are reported as ORs and 95% CIs.

#### Air pollution and cognition subdomains

Next, we tested the associations between each pollutant at age 10 and cognitive ability subdomains (crystallised ability, fluid ability and working memory) at age 12 with the use of linear regression models for exposure–outcome pairs that were significant in analyses for objective 1. We used a two-step procedure: we first tested an exposure–mediator association and a priori defined a p value lower than 0.15 to select appropriate mediators to include in our final multiple mediation analyses.^25^ We then estimated the IE, DE and total effect as above for each cognitive subdomain for significant exposure–outcome pairs. A higher p value for multiple mediators (p<0.15) was chosen to be inclusive of all potential mediators, which can be important when mediators are likely to be multicollinear or to ensure robustness of effect estimation of the a×b path while individual paths (a or b) may not be significant.

#### Sensitivity analyses

Following prior E-Risk air pollution analyses, we conducted four sensitivity analyses.^5 7 26^ (1) To address the high correlation between pollutants, we re-ran fully adjusted models and mediation analyses using one gaseous and one particulate exposure included simultaneously. (2) To assess the role of extreme levels of exposure, we dichotomised participants’ exposure into the top quartile versus the bottom three quartiles for each air pollutant. (3) To rule out the potential impact of moving address on air pollution exposure, we repeated full model analyses after removing participants who moved before age 10, or between ages 10 and 18. (4) Lastly, although we have included a comprehensive set of covariates, it is possible that residual confounding could remain. To assess the robustness of our results against the potential strength of any unmeasured confounding for the main association (objective 1), we calculated the E-value, a statistical reporting of the required effect estimate of any unmeasured confounding that would be required to nullify the association.^27^

## Findings

A total of 1969 participants (n=1034 female (52.5%)) of the age-18 wave (n=2066) had full data for exposures (n=73 missing) and mental health outcomes (n=24 missing). Prevalence and demographic characteristics of participants are reported in table 1. Exposure to PM_2.5_ ranged from 3.07 to 18.2 µg/m^3^, with 86% of participants residing in areas above the 2005 WHO guideline annual exposure limit of 10 µg/m^3^. NO_2_ exposure ranged from 2.6 to 57.9 µg/m^3^, with 10% of participants residing in areas above the then WHO guideline value of 40 µg/m^3^. PM_2.5_, NO_2_ and NO_x_ were all highly correlated (Pearson’s r>0.8) (online supplemental eTable 3).

**Table 1:** Descriptive statistics for air pollution exposures, mental health problems and covariates

Variables	N/mean (%/SD)	Missing (n)
	Total n=1969	
Assigned sex at birth		0
Male	935 (47.5)	
Female	1034 (52.5)	
Family social class composite at age 5		0
Low	668 (33.9)	
Medium	657 (33.4)	
High	644 (32.7)	
Neighbourhood deprivation (average across ages 5–10)		4
Wealthy achievers	390 (19.8)	
Urban prosperity	216 (11.0)	
Comfortably off	510 (26.0)	
Moderate means	364 (18.5)	
Hard pressed	485 (24.7)	
Urbanicity (average across ages 5–10)		2
Rural	383 (19.5)	
Urban city/own	940 (47.8)	
Major/minor conurbation	644 (32.7)	
Proportion of family members with any psychiatric disorder (assessed at age 12)	0.37 (0.27)	55
Ever a daily smoker—up to age 18		1
No	1456 (74.0)	
Yes	512 (26.0)	
Air Pollution—annual average at age 10 (median/IQR)		
Nitrogen dioxide (µg/m³)	24.7 (14.1)	0
Nitrogen oxides (µg/m³)	31.9 (22.8)	0
PM_10_ (µg/m³)	17.1 (2.7)	0
PM_2.5_ (µg/m³)	12.3 (2.0)	0
Mental health disorders—age 18		
Major depressive disorder		0
No	1570 (79.7)	
Yes	399 (20.3)	
Generalised anxiety disorder		0
No	1824 (92.6)	
Yes	145 (7.4)	
Attention-deficit/hyperactivity disorder		0
No	1809 (91.9)	
Yes	160 (8.1)	
Conduct disorder		0
No	1670 (84.8)	
Yes	299 (15.2)	
Cognition—age 12		
Overall cognitive ability (full-scale IQ)	100.2 (14.8)	55
Matrix reasoning scaled score (fluid ability)	9.5 (2.8)	55
Information scaled score (crystallised ability)	9.3 (3.2)	55
Digit span scaled score (working memory)	10.7 (3.3)	56

‘n’ reflects included participants only. All columns n (%) or mean (SD, aside from pollutant exposure in median (IQR)). Pollutants are presented prior to rescaling by IQR.

PM_10_, particulate matter size 10 µm or smaller; PM_2.5_, particulate matter size 2.5 µm or smaller.

### Associations between childhood air pollution exposures and early-adulthood mental health disorders

In models adjusting for all sociodemographic, neighbourhood and family covariates, only the association of NO_x_ and MDD remained statistically significant (OR=1.25, 95% CI 1.01 to 1.55). There were no statistically significant associations of any air pollutant exposure at age 10 with GAD or conduct disorder at age 18, and the unadjusted association between higher exposure to PM_2.5_ and greater odds of ADHD (OR=1.18, 95% CI 1.00 to 1.39) became non-significant following covariate adjustment (table 2).

**Table 2:** Associations between air pollutant exposures at age 10 (NO_2_, NO_x_, PM_2.5_ and PM_10_) and mental health disorders at age 18

Model	Major depressive disorder	Generalised anxiety disorder	ADHD	Conduct disorder
	OR (95% CI)	OR (95% CI)	OR (95% CI)	OR (95% CI)
Pollutant				
NO_2_				
Basic	1.15 (0.97 to 1.36)	1.15 (0.86 to 1.53)	1.22 (0.98 to 1.53)	1.09 (0.88 to 1.34)
Full	1.21 (0.96 to 1.51)	1.10 (0.76 to 1.60)	1.16 (0.86 to 1.56)	1.20 (0.89 to 1.60)
NO_x_				
Basic	1.17 (1.00 to 1.37)	1.15 (0.89 to 1.49)	1.19 (0.97 to 1.46)	1.09 (0.90 to 1.33)
Full	**1.25 (1.01 to 1.55)**	1.13 (0.81 to 1.59)	1.12 (0.85 to 1.47)	1.20 (0.92 to 1.57)
PM_2.5_				
Basic	1.02 (0.91 to 1.16)	1.01 (0.83 to 1.22)	**1.18 (1.00 to 1.39)**	1.01 (0.87 to 1.17)
Full	1.04 (0.91 to 1.18)	0.99 (0.81 to 1.20)	1.16 (0.97 to 1.39)	1.03 (0.87 to 1.21)
PM_10_				
Basic	1.04 (0.94 to 1.16)	1.15 (0.99 to 1.34)	1.10 (0.94 to 1.30)	1.02 (0.89 to 1.16)
Full	1.03 (0.93 to 1.15)	1.14 (0.97 to 1.34)	1.07 (0.90 to 1.28)	1.03 (0.89 to 1.19)

P values<0.05 are in bold font.

Basic=outcome and exposure, full=basic+assigned sex at birth, family socioeconomic status at age 5, neighbourhood deprivation (ages 5–10), urbanicity (ages 5–10), smoking status up to age 18 and the proportion of family members with a psychiatric history. ORs and 95% CI represent the increased odds of disorder status per interquartile range (μg/m3) increase in air pollutant levels. All associations are adjusted for the non-independence of twin observations with the CLUSTER command.

ADHD, attention-deficit/hyperactivity disorder; NO_2_, nitrogen dioxide; NO_x_, nitrogen oxides; PM_10_, particulate matter size 10µm or smaller; PM_2.5_, particulate matter size 2.5µm or smaller.

### Mediation of pollution–disorder associations by cognition

**Figure 1:**
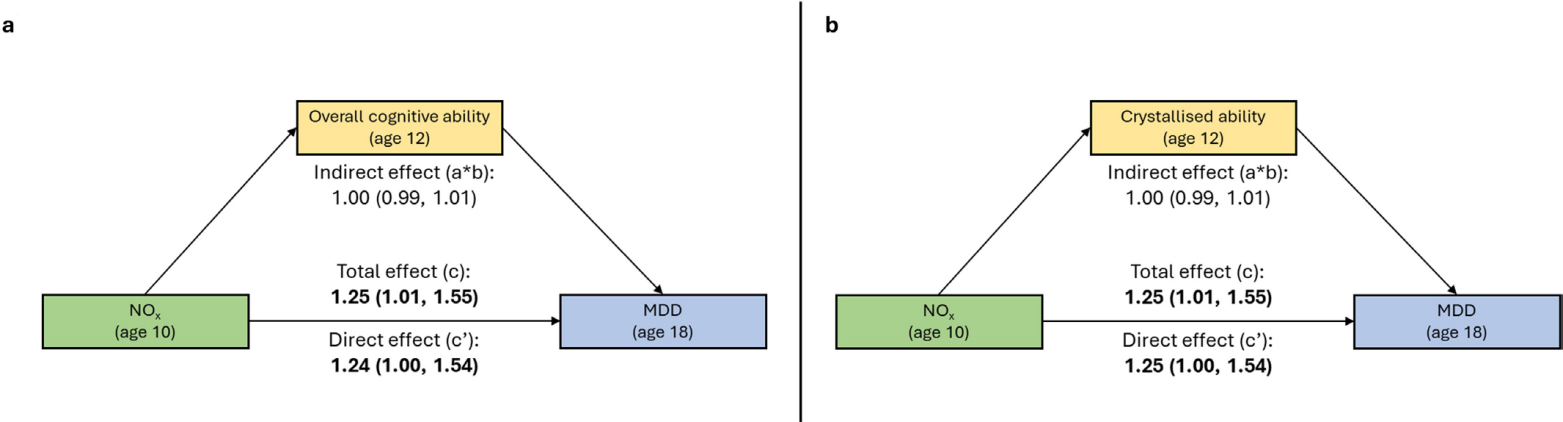
Mediation of associations between NO_x_ and MDD by overall cognition and crystallised ability at age 12. This represents the outcome of a generalised structural equation model as represented in directed acyclic graphs in online supplemental eFigure 3 and Methods. (a) Mediation (direct effect, indirect effect and total effect) via overall cognitive ability at age 12. (b) Mediation (direct effect, indirect effect and total effect) via the crystallised ability subdomain at age 12. ORs and 95% CI represent the increase in odds of disorder status per interquartile range (μg/m^3^) increase in air pollutant levels. Associations for full models are presented here, including exposure, outcome, assigned sex at birth, family socioeconomic status at age 5, neighbourhood deprivation (ages 5–10), urbanicity (ages 5–10), smoking status up to age 18 and the proportion of family members with a psychiatric history. P values <0.05 are in bold font. All associations are adjusted for the non-independence of twin observations with the CLUSTER command. MDD, major depressive disorder; NO_x_, nitrogen oxides.

No pollutant was associated with overall cognition, including NO_x_ (online supplemental eTable 4). Unsurprisingly, when we examined mediation of the association of NO_x_ and depression by overall cognition at age 12, we found no evidence of mediation (IE: OR=1.00, 95% CI 0.99 to 1.01; DE: OR=1.24, 95% CI 1.00 to 1.54) (figure 1). Only the crystallised ability subdomain was associated with NO_x_ exposure at age 10 (β=−0.19, 95% CI −0.42 to 0.04, p=0.11) (online supplemental eTable 4). PM_10_ was associated with working memory (β=0.15, 95% CI 0.00 to 0.30) but this was not included in the mediation analysis as per the analysis plan as there were no significant associations with PM_10_ in objective 1. There was no evidence of mediation of the NO_x_–depression association via crystallised ability (OR=1.00, 95% CI 0.99 to 1.01; DE: OR=1.25, 95% CI 1.00 to 1.54) (figure 1).

### Sensitivity analyses

In co-pollutant models of associations between air pollution exposure and mental health (online supplemental eTable 5), we found that higher levels of NO_x_ were significantly associated with greater odds of MDD even after controlling for PM_2.5_ (OR=1.56, 95% CI 1.12 to 2.17) or PM_10_ (OR=1.28, 95% CI 1.01 to 1.63). Generally, we saw effect size increases for NO_x_ and NO_2_ across disorders, aside from ADHD, where PM_2.5_ saw odds above 1. We further examined mediation of associations of NO_x_ with MDD in co-pollutant models and again found no evidence of mediation via overall cognition or crystallised ability (online supplemental eTables 6 and 7).

When examining the highest quartile of exposure compared with the lower three quartiles of exposure, only associations between NO_x_ and MDD were significant (OR=1.43, 95% CI 1.01 to 2.04), although some point estimates were inflated compared with the main analysis, suggesting non-linear effects (online supplemental eTable 8). However, we again saw no statistically significant mediation of the association of NO_x_ with MDD by overall cognition or crystallised ability in this ‘extremes’ analysis (IE via overall cognition: OR=1.00, 95% CI 0.99 to 1.01) (online supplemental eTable 9).

For non-movers between ages 5 and 10, we found no associations between exposure to any pollutants and any mental health outcome (online supplemental eTable 10). For non-movers between ages 10 and 18, we saw associations between NO_2_ (OR=1.62, 95% CI 1.08 to 2.42) and NO_x_ (OR=1.53, 95% CI 1.11 to 2.13) and conduct disorder (online supplemental eTable 11). We saw no mediation of NO_x_ and MDD by overall cognition or crystallised ability in non-movers between ages 5 and 10 or 10 and 18 (online supplemental eTable 12 and 13).

The observed OR of 1.25 for the association of NO_x_ and MDD had an E-value of 1.48, with a lower CI value of 1.08 (full model covariates are available in online supplemental eTable 14). The E-value is a theoretical value of the minimum strength of association that an unmeasured confounder would need to have on both the outcome and exposure to explain away the association. In our models, this E-value is below the strength of confounding by family psychiatric history or smoking but is still substantial. This suggests any unmeasured confounder would require a relatively strong association to nullify the association between NO_x_ and MDD, which increases our confidence in the robustness of the associations seen here.

## Discussion

In this two-decade prospective cohort study, we identified that exposure to ambient air pollution in late childhood, specifically NO_x_, was associated with a small increase in odds of MDD at age 18 after controlling for a wide range of confounders, including individual and neighbourhood-level sociodemographic factors. We then tested this association for potential mediation by cognitive factors and found that overall cognition and crystallised ability at age 12 did not mediate the association of NO_x_ with MDD in this sample.

Our results support a growing, if mixed evidence base that nitrogen-based gaseous exposures (NO_x_ and its constituent pollutant, NO_2_) may be associated with MDD. It builds on and replicates prior analysis in this cohort of associations with internalising symptoms^7^ and in exploratory analyses of MDD in E-Risk participants living in London,^5^ and in participants at higher risk of MDD.^26^ Findings in other studies for NO_2_/NO_x_ are not uniform across cohorts and exposure ages. An analysis of school-aged adolescents using the same exposure modelling as here found no association with NO_2_ exposure in late childhood/early adolescence and depression at ages 13–15, although this cohort was specific to London.^6^ The associations here between NO_x_ and MDD were robust in co-pollutant sensitivity models with particulate exposures, which may suggest a specificity of effect, but as pollutants in this sample were highly correlated, this makes it difficult to interpret. In the Avon Longitudinal Study of Parents and Children (ALSPAC) cohort, no evidence of associations between NO_2_ exposure and depression (between ages 13 and 18) was found when exposure occurred between ages 10 and 12 or earlier in childhood.^28^ It could be that our use of pollutant modelling at a relatively high resolution (20 m × 20 m) is estimating a pollutant fraction different to that used in the ALSPAC cohort (which was at 100 m × 100 m) and so might not be directly comparable, or that the association is specific to the whole NO_x_ exposure fraction, not just NO_2_. We decided not to adjust for multiple comparisons as we had strong a priori hypotheses, for which multiple testing correction can increase type 2 error. However, further replication is needed of this association as it could be a spurious finding.

We found no other associations in this cohort for any pollutant or outcome pair in fully adjusted models. In the ALSPAC cohort, PM_2.5_ was associated with later depression in young people.^28^ As this exposure was earlier in life than that examined here, it is possible that there is a degree of vulnerability to exposure during early life, although it is not possible in the current E-Risk cohort to examine pregnancy exposure. Alternatively, it could be due to variations in constituent parts of PM_2.5_ that may have different toxicology profiles between the cohorts, especially as ALSPAC participants are situated in just one region of the UK. The lack of significant associations here is also in contrast to prior analysis in the E-Risk cohort that found associations between exposure to NO_x_ and continuous measures of externalising disorder symptoms.^7^ As fully adjusted associations for both NO_2_ and NO_x_ with all outcomes examined here have a point estimate above 1, it is possible that this analysis was limited by sample size or by the sensitivity of diagnostic outcomes compared with continuous measures.

As exposure can be similar across periods of time in one area (and therefore, might be similar between earlier life exposure and age 10), we examined the moving status of participants, as moving may change the cumulative exposure patterns for individuals in this important period (ie, participants could move from high to low exposure). We found no associations for mental health disorders with any pollutant exposures in non-movers between the start of the study and age 10, though this may be partly due to the decreased sample size (n=1248). Interestingly, for participants who did not move between ages 10 and 18, we saw larger, statistically significant effect sizes between NO_x_, NO_2_ and conduct disorder compared with the whole sample. As we do not know if participants who did move ended up in areas of lower or higher exposure, it is only possible to tentatively suggest that moving may have been protective in this sample in relation to conduct disorder in young people.

Contrary to our original hypothesis, we found no mediation by overall cognition or crystallised ability at age 12 (we did not test for mediation via fluid ability or working memory, as these were not associated with NO_x_ exposure). There is extremely limited evidence for mediators between air pollution and mental health at any age.^9^ However, there is some evidence of cognition mediating associations between exposure to air pollution and mental health, although these studies have been limited to older adults.^14^ This, combined with the extensive literature on air pollution exposures and cognition,^11 13^ suggested that cognition may be a putative mediator. We expected that cognitive ability would represent an indirect mediator, on which pollution is acting through other physical processes that may influence neurodevelopment. Plausible mechanisms with evidence of action include neuroinflammatory and oxidative stress pathways and neural network disruption associated with higher exposure to some pollutants,^10^ including physical activity or markers of physical health changes, such as inflammatory biomarkers (eg, interleukin 6 or C reactive protein).^9^

The absence of mediation in this cohort may be due to several factors. First, we found no associations with any pollutant and overall cognition, and only with NO_x_-crystallised ability and PM_10_-working memory in the subdomains. This itself was unexpected compared with emerging evidence for particulate pollutants which suggests some association with cognition for earlier exposure, though generally is in line with the extremely limited evidence available for NO_x_/NO_2_.^13 29^ Second, although there is evidence that cognition could be associated with later mental health problems, depending on the particular cognitive domain and psychopathology, other studies report no associations.^30^ It is therefore likely that there was simply no association across the short exposure–outcome time window (ages 10, 12 and 18) between exposure, these specific measures of cognition and MDD.

Future research into potential mechanisms may wish to expand investigations to other cognitive domains such as processing speed, rumination and social cognition, or non-cognitive skills associated with mental health, including emotion regulation and distress tolerance. Future research should evaluate these, especially as there might be potential interventions that can support non-cognitive skills.

This study has several key strengths that include an in-depth, longitudinal dataset with comprehensive coverage of important socioeconomic and family-related confounders. Though residual confounding requires ongoing consideration, the E-value reported here for NO_x_ and MDD (1.48) would represent a relatively large unmeasured confounder that, considering the range of current measures included, leaves few, if any, plausible candidates that could meet this level. Beyond other studies in this age range, we also examined outcomes that were assessed using researcher interviews based on past-year symptomology, which allow for greater specificity for outcome measures compared with self-report questionnaires.

Nonetheless, some limitations should be considered. First, although the air pollution modelling employed here is at a comparatively high resolution (20 m × 20 m) and has strong performance statistics, we only used residential models, which may not accurately reflect exposure throughout participants’ everyday life (eg, travelling to and being at school). Second, we only used one time-point of air pollution data, which may mean some participants have a misclassification of their exposure prior to or after age 10, although our results are consistent within non-movers before and after age 10. Future research could incorporate longitudinal exposure models that may better capture longer-term exposure. Similarly, we only used one measure for each cognitive subdomain and only had access to single time-point measures for both mediator and outcome, which may increase error, reduce power and could lead to a bias towards the null. Thirdly, we were unable to control for the potentially confounding effect of other exposures such as noise.^6^ Lastly, E-Risk is broadly representative at inception of demographics in the constituent countries and may be generalisable to comparable high-income settings. However, it may not be generalisable to settings with much higher exposure profiles or different socio-economic situations. Finally, although we corrected statistically for the non-independence of twin observations, it would be important to replicate these findings in singleton cohorts, as it is possible there are gene-environment interactions among twins.

Overall, our results augment the growing evidence base identifying associations between air pollution exposure in early life and increased risk of mental health disorders in early adulthood, specifically between NO_x_ exposure and MDD, although we found that this was not mediated by cognition.

### Clinical implications

Air pollution may be a clinically relevant target for depression prevention. As we identified no mediation of the NO_x_-MDD association by cognition, as measured here at age 12, this suggests that other cognitive skills or non-cognitive deficits may underlie these associations (eg, emotion regulation). Future research may wish to explore other plausible candidate mechanisms, as policies specifically targeting cognition may not help reduce any fraction of mental ill health associated with higher air pollution exposure in young people. Overall, if the associations seen here are replicated, they suggest that policies working to reduce air pollution exposure, and possible targeted interventions (eg, emotional training), in young people may help reduce the risk of MDD.

## Supplementary material

10.1136/bmjment-2025-301864 online supplemental file 1

## Data Availability

Data are available on reasonable request.
