# Supplementary material for: Childhood exposure to particulate matter and nitrogen oxides and associations with mental health disorders in early adulthood: testing mediation by cognition in a UK longitudinal cohort study
Source: BMJ Ment Health. 2025 Sep 30;28(1):e301864. doi: 10.1136/bmjment-2025-301864 (PMC12496056; doi:10.1136/bmjment-2025-301864)
Supplement: online supplemental file 1 [file bmjment-28-1-s001.docx]

**Supplementary Materials**

**Canning *et al.* Childhood exposure to particulate matter and nitrogen oxides and associations with mental health disorders in early adulthood: testing mediation by cognition in a UK longitudinal cohort study**

**Contents**

[Supplementary methods 3](#_1a3ax8bkbhke)

[Sample 3](#_uosj6egqd0nt)

[eFigure 1. The E-Risk Study families’ addresses are a near-perfect match to the deciles of the UK Government’s Index of Multiple Deprivation. 5](#_w4zgsew34gvv)

[Changes to pre-registration of analyses 5](#_kesytzgwfy65)

[Measures 6](#_codblhwk9nqu)

[Air pollution 6](#_slfqm3odu7t3)

[Mental health 6](#_7ufltis41b5i)

[Family socioeconomic status 7](#_6u3v992e7ofx)

[Neighbourhood deprivation 8](#_inr36sm19npn)

[Urbanicity 8](#_fskphwqk8c3u)

[Covariate inclusion 9](#_pmvrchp3yft2)

[Multiple imputation by chained equations 9](#_io147foedkq0)

[References 10](#_s8n0x8nlbfwd)

[eTable 1: STROBE statement checklist 12](#_pc34jhs1giti)

[eTable 2: Performance statistics for the air pollution exposure estimates 16](#_fmkuy69fsi8r)

[eTable 3: Correlation coefficients between air pollution exposure estimates 17](#_c2jbtjyf1ibn)

[eTable 4: Associations between air pollutant exposure at age 10 (NO_2_, NO_x_, PM_2.5_ and PM_10_) and overall cognition and cognitive domains (fluid and crystallised ability, working memory) at age 12 18](#_tpi4kru6ambn)

[eTable 5: Co-pollutant analysis of associations between air pollutant exposures at age 10 (NO_2_, NO_x_, PM_2.5_ and PM_10_) and mental health disorders at age 18 19](#_j68m1ljb1p6n)

[eTable 6: Co-pollutant mediation analysis for general cognition between NO_X_ exposure at age 10 and MDD at age 18, including PM_2.5_ and PM_10_ 20](#_i0ro939tqe74)

[eTable 7: Co-pollutant mediation analysis for crystallised ability between NO_X_ exposure at age 10 and MDD at age 18, including PM_2.5_ and PM_10_ 21](#_etgwck9bzteg)

[eTable 8: Extremes analysis of associations between the top quartile versus any other quartile of air pollutant exposures at age 10 (NO_2_, NO_x_, PM_2.5_ and PM_10_) and mental health disorders at age 18 22](#_pmurfwkhrcj8)

[eTable 9: Mediation analysis of top quartile exposure versus any other quartile of NO_x_ at age 10, overall cognition or crystallised ability at age 12 and MDD at age 18 23](#_i3o9omt600py)

[eTable 10: Associations between air pollutant exposures at age 10 (NO_2_, NO_x_, PM_2.5_ and PM_10_) and mental health disorders at age 18 among those who did not move home address between ages 5 and 10 24](#_x56p7qnjfpme)

[eTable 11: Associations between air pollutant exposures at age 10 (NO_2_, NO_x_, PM_2.5_ and PM_10_) and mental health disorders at age 18 among those who did not move home address between ages 10 and 18 25](#_s4pupy94ghmt)

[eTable 12: Mediation analysis between NO_x_ exposure at age 10, cognition or crystallised ability at age 12 and MDD at age 18 for participants who did not move between ages 5-10 26](#_8vjh9yk3029z)

[eTable 13: Mediation analysis between NO_x_ exposure at age 10, cognition at age 12 and MDD at age 18 for participants who did not move between ages 10-18 27](#_bq6n7qlho6zl)

[eTable 14: Associations between exposure to NO_x_ at age 10 and MDD at age 18 with E-values and covariates 28](#_8tvfsoajxue7)

[eFigure 2 Participant flowchart for sample of E-Risk participants included in this study. 30](#_boajpqfg07ef)

[eFigure 3 Directed Acyclic Graph for exposure, outcome, mediators and confounders proposed in this study. 31](#_2ric0qgv7kew)

# Supplementary methods

## Sample

Participants were members of the Environmental Risk (E-Risk) Longitudinal Twin Study, which investigates how genetic and environmental factors shape children’s development. The sampling frame from which the E-Risk families were drawn was two consecutive birth cohorts (1994 and 1995) in a birth register of twins born in England and Wales.^1^ Of the 15,906 twin pairs born in these two years, 71% joined the register. The E-Risk Study probability sample was drawn using a high-risk stratification strategy. High-risk families were those in which the mother had her first birth when she was 20 years of age or younger. This sampling frame was used (1) to replace high risk families who were selectively lost to the register via non-response and (2) to ensure sufficient base rates of environmental risk factors. The high-risk sampling strategy resulted in a final sample in which one-third of Study mothers constitute a 160% oversample of mothers who were at high risk based on their young age at first birth (13–20 years), while the other two-thirds of Study mothers accurately represent all mothers in the general population (13–48 years) in England and Wales in 1994–95.

The Study sought a sample size of 1,100 families to allow for attrition in future years of the longitudinal study while retaining statistical power. An initial list of families who had same-sex twins was drawn from the register to target for home-visits, with a 10% oversample to allow for nonparticipation. Of the 1,203 families from the initial list who were eligible for inclusion, 1,116 (93%) participated in home-visit assessments when the twins were age 5 years, forming the base sample for the study (2,232 children): 4% of families refused, and 3% were lost to tracing or could not be reached after many attempts. All families are English speaking, and the majority (93.7%) are White.

Attrition has been minimal, and data has been successfully collected from 98% (at age 7 years), 96% (at age 10 years), 96% (at age 12 years), and most recently in 2012–2014, 93% of the original sample (at age 18 years). Home-visits at ages 5, 7, 10, and 12 years included face-to-face assessments with participants as well as their mother (or primary caregiver); the home-visit at age 18 included interviews only with the participants, and questionnaires completed by co-informants (caregivers and other family members). Each twin participant was assessed by a different interviewer. Most participants (71.4%; N=1475) lived at the same address between ages 12 and 18. In addition, adolescents who did move house tended to move to similar neighbourhoods: 87.0% of movers who lived in urban/intermediate neighbourhoods at age 12 also lived in urban/intermediate neighbourhoods at age 18.

There were 2,066 E-Risk participants who were assessed at age 18. The average age of the participants at the time of the assessment was 18.4 years (SD = 0.36); all interviews were conducted after the 18th birthday. There were no differences between those who did and did not take part at age 18 in terms of family socioeconomic status (SES) assessed when the cohort was initially defined (χ^2^ = 0.86, *p* = 0.65), age-5 IQ scores (*t* = 0.98, *p* = 0.33), or age-5 internalising or externalising behaviour problems (*t* = 0.40, *p* = 0.69 and *t* = 0.41, *p* = 0.68, respectively). E-Risk families are representative of UK households across the spectrum of neighbourhood-level deprivation: 25.6% of E-Risk families live in “wealthy achiever” neighbourhoods compared to 25.3% of households nation-wide; 5.3% vs 11.6% live in “urban prosperity” neighbourhoods; 29.6% vs 26.9% live in “comfortably off” neighbourhoods; 13.4% vs 13.9% live in “moderate means” neighbourhoods; and 26.1% vs 20.7% live in “hard-pressed” neighbourhoods.^2,3^ E-Risk underrepresents urban prosperity neighbourhoods because such households are likely to be childless.

eFigure 1 shows E-Risk families’ addresses are a near-perfect match to the deciles of the UK’s 2015 Lower-layer Super Output Area (LSOA) Index of Multiple Deprivation (IMD) which averages 1,500 residents; approximately 10% (dotted red line) of the E-Risk cohort fills each of the IMD’s 10% bands, indicating that the E-Risk cohort accurately represents the distribution of deprivation in the UK.
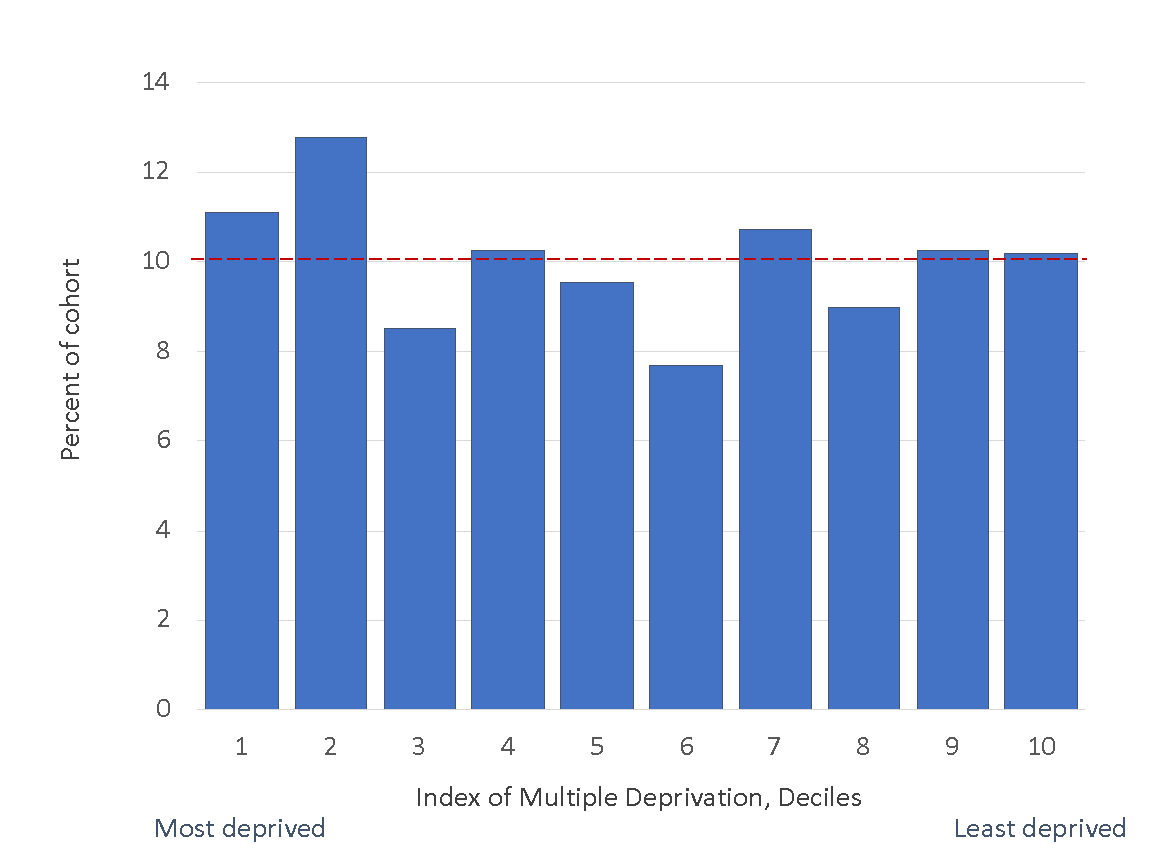


**eFigure 1. The E-Risk Study families’ addresses are a near-perfect match to the deciles of the UK Government’s Index of Multiple Deprivation.** *Note. The UK Ministry of Housing, Communities & Local Government Index of Multiple Deprivation is an official measure of relative deprivation for every LSOA small area (approximately 1,500 residents or 650 households each) in England.*

## Changes to pre-registration of analyses

The four amendments to the analysis plan were the removal of bootstrapping for the mediation analysis as this was a duplication of robust standard errors generated using the CLUSTER command, the addition of urbanicity to the statistical models and directed acyclic graph to better capture neighbourhood-level confounding, the reporting of associations between exposures at age 10 and overall cognition at age 12 to improve transparency and the inclusion of the imputed mediators in mediation analysis.

## Measures

### Air pollution

Exposure estimates were modelled and linked to the latitude-longitude coordinates of participants’ home addresses at age 10. Pollution estimates were modelled using CMAQ-urban, which is a coupled regional chemical transport model and street-scale dispersion model. CMAQ-urban uses a new generation of road traffic emissions inventory in the UK to model air quality down to individual streets, providing hourly estimates of pollutants at 20x20 metre grid points throughout the UK (i.e., address-level). Full details on the creation and validation of this model have been described previously.^4,5^ Briefly, modelled estimates were compared to readings from the Automatic Urban and Rural Network (AURN) and London Air Quality Network (LAQN) (which has a mix of rural, urban, roadside, kerbside and industrial sites) measurement programmes. The model performs well (Supplementary eTable 2). The model provides slight underestimates for NO_2_ (by 1.02μg/m^3^ or 2%), NO_X_ (by 10.90μg/m^3^ or 11%), PM_2.5_ (by 0.70μg/m^3^ or 4%) and PM_10_ (by 2.72μg/m^3^ or 10%). R and RMSE values suggest spatial variations for predicted NO_2,_ and NO_x_ values are accurate, though less so for PM_10_ (RMSE=6.49, r=0.54) and PM_2.5_ (RMSE=3.54, r=0.66).

### Mental health

The interview began with four screening questions to identify participants who had experienced at least 2 weeks of persistent low mood, anhedonia, or irritability in the past year, or those who had been prescribed medication for depression. Participants who answered positively to any of the screening items were asked a further 24 questions designed to map onto the nine symptom­ criteria of a major depressive episode specified in the Diagnostic and Statistical Manual of Mental Disorders, Fourth Edition (DSM-IV). To identify participants with clinically significant depression we used a diagnostic cut-off based on the presence of at least five symptom-criteria plus interference in daily functioning. Generalised anxiety disorder (GAD) diagnosis was defined by DSM-IV criteria. Participants were asked if they had a period of at least a month of persistent anxious feelings or worried most days more than others, about 2 or more different factors. If so, they were asked further follow up questions about symptoms, for which a GAD diagnosis was given if they had anxiety or worry for three or more of the following symptoms (restlessness or feeling keyed up or on edge; being easily fatigued; difficulty concentrating or mind going blank; irritability; muscle tension; sleep disturbance (difficulty falling or staying asleep, or restless unsatisfying sleep)) and these interfered with daily functioning. Conduct problems were defined as a score of five or more out of 13 items (scored yes/no): items included the use of weapons (e.g., “have you used a weapon on someone like a knife, piece of wood, or baseball bat?”), truancy (e.g., “do you sometimes skip school when you shouldn’t?”), and stealing (e.g., “have you stolen something while nobody was looking?”). At age 18, an additional 20 items were asked around age-related antisocial behaviours such as “have you stolen a car or motorcycle?” or “have you sold hard drugs?”. For ADHD, diagnoses were made based on DSM-V criteria. Participants had to endorse 5 or more inattentive and/or five or more hyperactivity-impulsivity symptoms to receive a diagnosis. These symptoms were required to interfere with participants’ life at home, or with family and friends and at school or work to meet impairment and pervasiveness criteria.

### Family socioeconomic status

Family SES was measured via a standardised composite of total household income, highest maternal/paternal education, and highest maternal/paternal occupation when children were aged 5. These three indicators were highly correlated (r’s ranged from 0.57-0.67, p’s<.05) and loaded significantly onto one latent factor (factor loadings=0.80, 0.70, and 0.83 for income, education, and occupation, respectively). This latent variable was then categorised into tertiles (i.e., low-, medium-, and high-SES) to improve interpretability of the score.^6^

### Neighbourhood deprivation

Neighbourhood deprivation was included as a geodemographic discriminator that used over 400 census variables from Great Britain (CACI Information Services; <http://www.caci.co.uk/>).^3^ Classifications (ranging from “Wealthy Achiever” (coded 1) to “Urban Prosperity” (coded 2), to “Comfortably Off” (coded 3), to “Moderate Means” (coded 4), to “Hard Pressed” (coded 5) neighbourhoods) were linked to home postcodes at ages 5, 7, and 10, and then averaged and rounded to the nearest whole number across ages 5–10.

### Urbanicity

Urbanicity was derived from the UK Office for National Statistics (ONS) Rural-Urban Definition for Small Area Geographies (RUC2011) classifications.^6^ The ONS classifications utilised 2011 census data and were designed for application to small geostatistical units (e.g. Output Areas). Briefly, RUC2011 was created by laying a grid of hectare cells (100m^2^) over England and Wales. Postcode addresses were assigned to cells, and residential densities were then calculated for increasing radii around each cell, providing each residential property with a density profile. This was combined with Output Area and contextual data, allowing each settlement to be assigned to one of ten urbanicity categories (Rural categories: sparse/non-sparse hamlets and isolated dwellings, sparse/non-sparse villages, sparse/non-sparse rural town and fringe; Urban categories: sparse/non-sparse city and town, and minor/major conurbations [conurbations are densely populated, large urban regions resulting from the expansion and coalescence of adjacent cities and towns]). ONS urbanicity scores were then assigned to every E-Risk family via the family’s postcode when children were aged 5, 7 and 10, and then averaged across ages 5-10 and rounded to the nearest number. Given the low numbers within some rural categories, urbanicity was collapsed into three levels (1: “rural” = all rural categories [20.3% of participants at age 10]; 2: “intermediate” = urban cities and towns [47.6% of participants at age 10]; and 3: “urban” = minor/major conurbations [32.1% of participants at age 10]). E-Risk participants are nationally-representative in terms of ONS urbanicity classifications; 32.1% of E-Risk participants lived in urban settings at age 10 compared to 36.1% nationwide; 47.6% versus 45.0% lived in intermediate settings; and 20.3% versus 18.9% lived in rural settings.

### Covariate inclusion

Assigned sex at birth was included due to differences in mental health, cognition^7^ and potentially with air pollution exposure at age 10.^8^ Family socioeconomic status (SES) was included as lower socioeconomic status is associated with poorer mental health, cognition^9^ and higher air pollution exposure.^10,11^ Neighbourhood deprivation was included to capture wider neighbourhood level contextual factors, and higher deprivation and urbanicity are associated with higher air pollution exposure and poorer mental health and cognition.^12–14^ We included an urbanicity measure, for associations with exposure^15^ and outcome or mediator.^16,17^ Family psychiatric history was included as it is associated with mental health problems and poorer cognition in offspring,^18,19^ and could be associated with air pollution, particularly through family SES. Smoking was included to minimise misclassification of air pollution exposure through inhalation of tobacco smoke, and as a potential confounder for cognition^20^ and mental health.^21^

## Multiple imputation by chained equations

We imputed missing covariate data for those with complete mental health disorders and air pollution exposure data using “mi impute chained” in Stata 18.0 MP. Imputed variables included family psychiatric history (N missing = 55), neighbourhood deprivation at ages 5, 7, and 10 (N=33, 28 and 12, respectively), urbanicity at ages 5, 7, and 10 (N=93, 44 and 14 respectively), smoking (N=1) and overall cognition, fluid and crystallised ability (all N=55) and working memory (N=56). Complete variables were included in the imputation (family id for twins, assigned sex at birth, air pollution exposure, mental health outcomes, family socioeconomic status at age 5). We imputed 15 datasets using a random seed of 2468. Imputation resulted in a sample size of N=1969 for mental health models (versus N=1,907 for complete case analyses).

## References

1 Moffitt TE, the E-Risk Study Team. Teen-aged mothers in contemporary Britain. *J Child Psychol Psychiatry*. 2002;43:727–42. doi: 10.1111/1469-7610.00082

2 CACI Information Services. ACORN user guide. London, United Kingdom: CACI 2006.

3 Caspi A, Taylor A, Moffitt TE, *et al.* Neighborhood deprivation affects children’s mental health: Environmental risks identified in a genetic design. *Psychol Sci*. 2000;11:338–42. doi: 10.1111/1467-9280.00267

4 Beevers SD, Kitwiroon N, Williams ML, *et al.* One way coupling of CMAQ and a road source dispersion model for fine scale air pollution predictions. *Atmos Environ*. 2012;59:47–58. doi: 10.1016/j.atmosenv.2012.05.034

5 Carslaw D. Defra urban model evaluation analysis - Phase 1. Report to Defra, Environmental Research Group. 2011. Available at https://uk-air.defra.gov.uk/library/reports?report_id=654. Accessed on 08/09/2025

6 Trzesniewski KH, Moffitt TE, Caspi A, *et al.* Revisiting the association between reading achievement and antisocial behavior: New evidence of an environmental explanation from a twin study. *Child Dev*. 2006;77:72–88. doi: 10.1111/j.1467-8624.2006.00857.x

7 Tonne C, Milà C, Fecht D, *et al.* Socioeconomic and ethnic inequalities in exposure to air and noise pollution in London. *Environ Int*. 2018;115:170–9. doi: 10.1016/j.envint.2018.03.023

8 Reiss F. Socioeconomic inequalities and mental health problems in children and adolescents: A systematic review. *Soc Sci Med*. 2013;90:24–31. doi: 10.1016/j.socscimed.2013.04.026

9 Jonsson KR, Vartanova I, Södergren M. Ethnic variations in mental health among 10–15-year-olds living in England and Wales: The impact of neighbourhood characteristics and parental behaviour. *Health Place*. 2018;51:189–99. doi: 10.1016/j.healthplace.2018.03.010

10 Gray NR, Lewis AC, Moller SJ. Deprivation based inequality in NO _x_ emissions in England. *Environ Sci Adv*. 2023;2:1261–72. doi: 10.1039/D3VA00054K

11 McCulloch A, Joshi HE. Neighbourhood and family influences on the cognitive ability of children in the British National Child Development Study. *Soc Sci Med*. 2001;53:579–91. doi: 10.1016/S0277-9536(00)00362-2

12 Milojevic A, Niedzwiedz CL, Pearce J, *et al.* Socioeconomic and urban-rural differentials in exposure to air pollution and mortality burden in England. *Environ Health*. 2017;16:104. doi: 10.1186/s12940-017-0314-5

13 Xu C, Miao L, Turner D, *et al.* Urbanicity and depression: A global meta-analysis. *J Affect Disord*. 2023;340:299–311. doi: 10.1016/j.jad.2023.08.030

14 Bijnens EM, Derom C, Thiery E, *et al.* Residential green space and child intelligence and behavior across urban, suburban, and rural areas in Belgium: A longitudinal birth cohort study of twins. *PLOS Med*. 2020;17:e1003213. doi: 10.1371/journal.pmed.1003213

15 Van Dijk MT, Murphy E, Posner JE, *et al.* Association of Multigenerational Family History of Depression With Lifetime Depressive and Other Psychiatric Disorders in Children: Results from the Adolescent Brain Cognitive Development (ABCD) Study. *JAMA Psychiatry*. 2021;78:778. doi: 10.1001/jamapsychiatry.2021.0350

16 MacKenzie LE, Howes Vallis E, Rempel S, *et al.* Cognition in offspring of parents with psychotic and non-psychotic severe mental illness. *J Psychiatr Res*. 2020;130:306–12. doi: 10.1016/j.jpsychires.2020.08.019

17 Jacobsen LK, Krystal JH, Mencl WE, *et al.* Effects of smoking and smoking abstinence on cognition in adolescent tobacco smokers. *Biol Psychiatry*. 2005;57:56–66. doi: 10.1016/j.biopsych.2004.10.022

18 Stansfeld S, Clark C, Bebbington P, *et al.* Chapter 2: Common mental disorders. In: McManus S, Bebbington P, Jenkins R, *et al.*, eds. *Mental health and wellbeing in England: Adult Psychiatric Morbidity Survey 2014*. Leeds: NHS Digital 2016.

# eTable 1: STROBE statement checklist

|  | **Item No** | **Recommendation** | **Detail** |
| --- | --- | --- | --- |
| **Title and abstract** | 1 | (*a*) Indicate the study’s design with a commonly used term in the title or the abstract | “longitudinal cohort study” is stated in the title on page 1 of the main manuscript |
|  |  | (*b*) Provide in the abstract an informative and balanced summary of what was done and what was found | This is provided in the abstract on page 2 of the main manuscript |
| **Introduction** | | |  |
| Background/rationale | 2 | Explain the scientific background and rationale for the investigation being reported | This is provided in the background section of the main manuscript on page 4 |
| Objectives | 3 | State specific objectives, including any prespecified hypotheses | Objectives and hypotheses clearly stated in background section on pages 4-5 of the main manuscript |
| **Methods** | | |  |
| Study design | 4 | Present key elements of study design early in the paper | Detailed in methods section on page 5 of the main manuscript and supplementary methods (pages 3-5 of supplement). |
| Setting | 5 | Describe the setting, locations, and relevant dates, including periods of recruitment, exposure, follow-up, and data collection | Detailed in methods section on page 5 of the main manuscript and supplementary methods (pages 3-5 of supplement). |
| Participants | 6 | (*a*) Give the eligibility criteria, and the sources and methods of selection of participants. Describe methods of follow-up | Detailed in methods section on page 5 of the main manuscript and supplementary methods (pages 3-5 of supplement). |
|  |  | (*b*) For matched studies, give matching criteria and number of exposed and unexposed | n/a |
| Variables | 7 | Clearly define all outcomes, exposures, predictors, potential confounders, and effect modifiers. Give diagnostic criteria, if applicable | Detailed in methods section on pages 5-6 of the main manuscript. Extra detail in supplementary methods (pages 6-9 of supplement). |
| Data sources/ measurement | 8* | For each variable of interest, give sources of data and details of methods of assessment (measurement). Describe comparability of assessment methods if there is more than one group | Detailed in methods section on pages 5-6 of the main manuscript. |
| Bias | 9 | Describe any efforts to address potential sources of bias | Attrition is a large source of bias and has been minimal in this cohort (7%), detailed in methods section on pages 5 of the main manuscript, with reported differences between those retained vs lost in Supplementary Methods 1 (pages 3-4 of supplement). |
| Study size | 10 | Explain how the study size was arrived at | We included all participants who had full exposure and outcome information, as detailed in Methods section on page 6 of the main manuscript. |
| Quantitative variables | 11 | Explain how quantitative variables were handled in the analyses. If applicable, describe which groupings were chosen and why | Detailed in methods section on pages 6-7 of the main manuscript. |
| Statistical methods | 12 | (*a*) Describe all statistical methods, including those used to control for confounding | Provided on pages 6-7 of the main manuscript. |
|  |  | (*b*) Describe any methods used to examine subgroups and interactions | No subgroup or interaction analyses |
|  |  | (*c*) Explain how missing data were addressed | Provided on page 6 of the main manuscript and in Supplementary Methods (page 9 of supplement). |
|  |  | (*d*) If applicable, explain how loss to follow-up was addressed | Not addressed as minimal (detailed on page 5 of the main manuscript). |
|  |  | (*e*) Describe any sensitivity analyses | Provided on page 7 of the main manuscript. |
| **Results** | | |  |
| Participants | 13* | (a) Report numbers of individuals at each stage of study—eg numbers potentially eligible, examined for eligibility, confirmed eligible, included in the study, completing follow-up, and analysed | Provided on pages 5 and 8 of the main manuscript and in a participant flowchart in Supplementary eFigure 2 (page 30 of supplement). |
|  |  | (b) Give reasons for non-participation at each stage | Full details in Supplementary Methods (pages 3-4 of supplement) |
|  |  | (c) Consider use of a flow diagram | Provided in Supplementary eFigure 2 on page 30 of Supplementary Materials. |
| Descriptive data | 14* | (a) Give characteristics of study participants (eg demographic, clinical, social) and information on exposures and potential confounders | Full details in Supplementary Methods (pages 3-9 of supplement) and in Table 1 on pages 8-9 of main manuscript. |
|  |  | (b) Indicate number of participants with missing data for each variable of interest | Provided in Table 1 on pages 8-9 of main manuscript. |
|  |  | (c) Summarise follow-up time (eg, average and total amount) | n/a |
| Outcome data | 15* | Report numbers of outcome events or summary measures over time | Reported % of participants with each outcome in results section 1 (page 8), and in Table 1 on pages 8-9 of main manuscript. |
| Main results | 16 | (*a*) Give unadjusted estimates and, if applicable, confounder-adjusted estimates and their precision (eg, 95% confidence interval). Make clear which confounders were adjusted for and why they were included | Justification in methods on pages 6-7 of main manuscript and precision given with 95% CIs for unadjusted/full models in text on pages 9-10 and in Table 2 on page 10 of main manuscript. |
|  |  | (*b*) Report category boundaries when continuous variables were categorized | n/a |
|  |  | (*c*) If relevant, consider translating estimates of relative risk into absolute risk for a meaningful time period | n/a |
| Other analyses | 17 | Report other analyses done—eg analyses of subgroups and interactions, and sensitivity analyses | Sensitivity analyses reported on pages 9-10 of main manuscript (with full results presented in supplementary tables on pages 19-27 of supplement). |
| **Discussion** | | |  |
| Key results | 18 | Summarise key results with reference to study objectives | Included in discussion on pages 11-12 of the main manuscript. |
| Limitations | 19 | Discuss limitations of the study, taking into account sources of potential bias or imprecision. Discuss both direction and magnitude of any potential bias | Detailed on pages 12-13 of the main manuscript. |
| Interpretation | 20 | Give a cautious overall interpretation of results considering objectives, limitations, multiplicity of analyses, results from similar studies, and other relevant evidence | Provided on pages 12-13 of the main manuscript. |
| Generalisability | 21 | Discuss the generalisability (external validity) of the study results | Detailed on pages 12-13 of the main manuscript. |
| **Other information** | | |  |
| Funding | 22 | Give the source of funding and the role of the funders for the present study and, if applicable, for the original study on which the present article is based | Provided on page 14 of the main manuscript. |

# eTable 2: Performance statistics for the air pollution exposure estimates

| **Pollutant** | **Year** | **Assessment age** | **Number of data points** | **Observed mean (μg/m^3^)** | **Modelled mean (μg/m^3^)** | **FAC2** | **MB (μg/m^3^)** | **NMB** | **RMSE (μg/m^3^)** | **r** |
| --- | --- | --- | --- | --- | --- | --- | --- | --- | --- | --- |
| NO_2_ | 2004 | 10 | 184 | 42.70 | 41.67 | 0.98 | -1.02 | -0.02 | 13.49 | 0.78 |
| NO_x_ | 2004 | 10 | 189 | 97.72 | 86.74 | 0.92 | -10.90 | -0.11 | 42.76 | 0.82 |
| PM_2.5_ | 2004 | 10 | 8 | 17.48 | 16.78 | 1 | -0.70 | -0.04 | 3.54 | 0.66 |
| PM_10_ | 2004 | 10 | 154 | 26.24 | 23.20 | 0.99 | -2.72 | -0.10 | 6.49 | 0.54 |

*NO_2_ = Nitrogen dioxide, NO_x_ = Nitrogen oxides, PM_2.5_= particulate matter size 2.5µm or smaller, PM_10_ = particulate matter size 10µm or smaller. FAC2 = fraction of predictions within a factor of two; MB = mean bias; NMB = normalised mean bias; RMSE = root mean squared error; r = correlation coefficient between observed and modelled.*

# eTable 3: Correlation coefficients between air pollution exposure estimates

|  | NO_2_ | NO_x_ | PM_2.5_ | PM_10_ |
| --- | --- | --- | --- | --- |
| NO_2_ | - |  |  |  |
| NO_x_ | 0.98 | - |  |  |
| PM_2.5_ | 0.84 | 0.82 | - |  |
| PM_10_ | 0.44 | 0.44 | 0.57 | - |

*NO_2_ = Nitrogen dioxide, NO_x_ = Nitrogen oxides, PM_2.5_= particulate matter size 2.5µm or smaller, PM_10_ = particulate matter size 10µm or smaller.*

# eTable 4: Associations between air pollutant exposure at age 10 (NO_2_, NO_x_, PM_2.5_ and PM_10_) and overall cognition and cognitive domains (fluid and crystallised ability, working memory) at age 12

|  | Overall cognition | | Fluid ability | | Crystallised ability | | Working memory | |
| --- | --- | --- | --- | --- | --- | --- | --- | --- |
| Pollutant | β | 95% CI | β | 95% CI | β | 95% CI | β | 95% CI |
| NO_2_ | -0.20 | -1.33, 0.93 | -0.05 | -0.25, 0.14 | -0.17 | -0.42, 0.07 | 0.12 | -0.12, 0.37 |
| NO_x_ | -0.19 | -1.27, 0.90 | -0.04 | -0.23, 0.15 | **-0.19** | **-0.42, 0.04** | 0.13 | -0.10, 0.37 |
| PM_2.5_ | 0.19 | -0.58, 0.96 | 0.00 | -0.13, 0.13 | 0.03 | -0.13, 0.19 | 0.07 | -0.10, 0.24 |
| PM_10_ | 0.40 | -0.30, 1.11 | 0.02 | -0.11, 0.16 | 0.03 | -0.12, 0.18 | **0.15** | **0.00, 0.30** |

*p < 0.15 in bold font. NO_2_ = Nitrogen dioxide. NO_x_ = Nitrogen oxides, PM_2.5_= particulate matter size 2.5µm or smaller. PM_10_= particulate matter size 10µm or smaller. Associations are between exposure and overall cognition or cognitive subdomains unadjusted for any covariates. Beta (β) coefficient and 95% confidence intervals (95% CI) represent the mean difference in score per interquartile range (μg/m^3^) increase in air pollutant levels. All associations are adjusted for non-independence of twin observations with the CLUSTER command.*

# eTable 5: Co-pollutant analysis of associations between air pollutant exposures at age 10 (NO_2_, NO_x_, PM_2.5_ and PM_10_) and mental health disorders at age 18

|  | Major depressive disorder | | Generalised anxiety disorder | | ADHD | | Conduct disorder | |
| --- | --- | --- | --- | --- | --- | --- | --- | --- |
| Co-pollutants | OR | 95% CI | OR | 95% CI | OR | 95% CI | OR | 95% CI |
| NO_2_ | **1.52** | **1.04, 2.22** | 1.43 | 0.75, 2.70 | 0.81 | 0.47, 1.40 | 1.53 | 0.94, 2.50 |
| PM_2.5_ | 0.86 | 0.69, 1.06 | 0.84 | 0.60, 1.19 | 1.28 | 0.93, 1.76 | 0.85 | 0.64, 1.12 |
|  |  |  |  |  |  |  |  |  |
| NO_2_ | 1.23 | 0.95, 1.58 | 0.97 | 0.65, 1.47 | 1.11 | 0.80, 1.55 | 1.22 | 0.88, 1.68 |
| PM_10_ | 0.98 | 0.87, 1.12 | 1.15 | 0.96, 1.37 | 1.04 | 0.85, 1.28 | 0.98 | 0.83, 1.16 |
|  |  |  |  |  |  |  |  |  |
| NO_x_ | **1.56** | **1.12, 2.17** | 1.42 | 0.84, 2.37 | 0.81 | 0.50, 1.29 | 1.47 | 0.98, 2.20 |
| PM_2.5_ | 0.84 | 0.70, 1.02 | 0.84 | 0.63, 1.13 | 1.29 | 0.96, 1.73 | 0.86 | 0.67, 1.11 |
|  |  |  |  |  |  |  |  |  |
| NO_x_ | **1.28** | **1.01, 1.63** | 1.01 | 0.69, 1.48 | 1.07 | 0.78, 1.47 | 1.23 | 0.92, 1.66 |
| PM_10_ | 0.97 | 0.85, 1.10 | 1.14 | 0.95, 1.36 | 1.05 | 0.86, 1.29 | 0.97 | 0.82, 1.15 |

*NO_2_ = Nitrogen dioxide, NO_x_ = Nitrogen oxides, PM_2.5_= particulate matter size 2.5µm or smaller, PM_10_ = particulate matter size 10µm or smaller. MDD = Major Depressive Disorder. P-value <0.05 in bold. Associations with full models are presented here, including exposure, outcome, assigned sex at birth, family socio-economic status at age 5, neighbourhood deprivation (ages 5-10), urbanicity (ages 5-10), smoking status up to age 18, and the proportion of family members with a psychiatric history. Odds ratios (OR) and 95% confidence intervals (95% CI) represent the increased odds of disorder status per interquartile range (μg/m^3^) increase in air pollutant levels. All associations are adjusted for non-independence of twin observations with the CLUSTER command.*

# eTable 6: Co-pollutant mediation analysis for general cognition between NO_X_ exposure at age 10 and MDD at age 18, including PM_2.5_ and PM_10_

|  | Direct effect | | Indirect effect | | Total effect | |
| --- | --- | --- | --- | --- | --- | --- |
| Co-pollutants | OR | 95% CI | OR | 95% CI | OR | 95% CI |
| NO_x_ | **1.55** | **1.11, 2.16** | 1.00 | 0.99, 1.02 | **1.56** | 1.12, 2.17 |
| PM_2.5_ | 0.84 | 0.70, 1.02 | 1.00 | 0.99, 1.00 | 0.84 | 0.70, 1.02 |
|  |  |  |  |  |  |  |
| NO_x_ | **1.28** | **1.01, 1.63** | 1.00 | 0.99, 1.01 | **1.28** | **1.01, 1.64** |
| PM_10_ | 0.96 | 0.85, 1.10 | 1.00 | 1.00, 1.01 | 0.97 | 0.85, 1.10 |

*P-value <0.05 in bold. NO_x_ = Nitrogen oxides, PM_2.5_= particulate matter size 2.5µm or smaller. PM_10_= particulate matter size 10µm or smaller. MDD = Major Depressive Disorder. Associations show the indirect effect (a*b), the direct effect (c'), and the total effect (c = a*b + c') of air pollution exposure on mental health disorders, with full models including exposure, outcome, assigned sex at birth, family socio-economic status at age 5, neighbourhood deprivation (ages 5-10), urbanicity (ages 5-10), smoking status up to age 18, and the proportion of family members with a psychiatric history. Odds ratios (OR) and 95% confidence intervals (95% CI) represent the increased odds of disorder status per interquartile range (μg/m^3^) increase in air pollutant levels. All associations are adjusted for non-independence of twin observations with the CLUSTER command. The direct effect represents the direct effect of exposure on MDD odds, independent of overall cognition at age 12. The indirect effect represents the association between exposure and MDD via cognition.*

# eTable 7: Co-pollutant mediation analysis for crystallised ability between NO_X_ exposure at age 10 and MDD at age 18, including PM_2.5_ and PM_10_

|  | Direct effect | | Indirect effect | | Total effect | |
| --- | --- | --- | --- | --- | --- | --- |
| Co-pollutants | OR | 95% CI | OR | 95% CI | OR | 95% CI |
| NO_x_ | **1.56** | **1.12, 2.17** | 1.00 | 0.98, 1.01 | **1.55** | **1.12, 2.16** |
| PM_2.5_ | 0.84 | 0.69, 1.02 | 1.00 | 0.99, 1.01 | 0.84 | 0.70, 1.03 |
|  |  |  |  |  |  |  |
| NO_x_ | **1.28** | **1.01, 1.64** | 1.00 | 0.99, 1.01 | **1.28** | **1.01, 1.64** |
| PM_10_ | 0.96 | 0.85, 1.10 | 1.00 | 1.00, 1.01 | 0.97 | 0.85, 1.10 |

*P-value <0.05 in bold. NO_x_ = Nitrogen oxides, PM_2.5_= particulate matter size 2.5µm or smaller. PM_10_= particulate matter size 10µm or smaller. MDD = Major Depressive Disorder. Associations show the indirect effect (a*b), the direct effect (c'), and the total effect (c = a*b + c') of air pollution exposure on mental health disorders, with full models including exposure, outcome, assigned sex at birth, family socio-economic status at age 5, neighbourhood deprivation (ages 5-10), urbanicity (ages 5-10), smoking status up to age 18, and the proportion of family members with a psychiatric history. Odds ratios (OR) and 95% confidence intervals (95% CI) represent the increased odds of disorder status per interquartile range (μg/m^3^) increase in air pollutant levels. All associations are adjusted for non-independence of twin observations with the CLUSTER command. The direct effect represents the direct effect of exposure on MDD odds, independent of crystallised ability at age 12. The indirect effect represents the association between exposure and MDD via crystallised ability.*

# eTable 8: Extremes analysis of associations between the top quartile versus any other quartile of air pollutant exposures at age 10 (NO_2_, NO_x_, PM_2.5_ and PM_10_) and mental health disorders at age 18

|  | Major depressive disorder | | Generalised anxiety disorder | | ADHD | | Conduct disorder | |
| --- | --- | --- | --- | --- | --- | --- | --- | --- |
| Pollutant | OR | 95% CI | OR | 95% CI | OR | 95% CI | OR | 95% CI |
| NO_2_ | 1.16 | 0.82, 1.65 | 1.43 | 0.81, 2.50 | 1.16 | 0.73, 1.85 | 1.45 | 0.91, 2.32 |
| NO_x_ | **1.43** | **1.01, 2.04** | 1.34 | 0.78, 2.32 | 1.03 | 0.65, 1.62 | 1.45 | 0.94, 2.24 |
| PM_2.5_ | 1.33 | 0.96, 1.84 | 1.08 | 0.66, 1.78 | 1.07 | 0.68, 1.69 | 0.90 | 0.60, 1.35 |
| PM_10_ | 0.91 | 0.69, 1.21 | 1.06 | 0.70, 1.62 | 0.96 | 0.64, 1.44 | 0.90 | 0.63, 1.28 |

*P-value <0.05 in bold. NO_2_ = Nitrogen dioxide, NO_x_ = Nitrogen oxides, PM_2.5_= particulate matter size 2.5µm or smaller, PM_10_ = particulate matter size 10µm or smaller. MDD = Major Depressive Disorder. Associations with full models are presented here, including exposure, outcome, assigned sex at birth, family socio-economic status at age 5, neighbourhood deprivation (ages 5-10), urbanicity (ages 5-10), smoking status up to age 18, and the proportion of family members with a psychiatric history. Odds ratios (OR) and 95% confidence intervals (95% CI) represent the increased odds of disorder status between the top quartile of exposure and the bottom three quartiles. All associations are adjusted for non-independence of twin observations with the CLUSTER command.*

# eTable 9: Mediation analysis of top quartile exposure versus any other quartile of NO_x_ at age 10, overall cognition or crystallised ability at age 12 and MDD at age 18

|  | Direct effect | | Indirect effect | | Total effect | |
| --- | --- | --- | --- | --- | --- | --- |
| Mediator | OR | 95% CI | OR | 95% CI | OR | 95% CI |
| Overall cognition | **1.43** | **1.00, 2.03** | 1.00 | 0.99, 1.01 | **1.43** | **1.01, 2.04** |
| Crystallised ability | **1.44** | **1.01, 2.05** | 0.99 | 0.98, 1.01 | **1.43** | **1.01, 2.04** |

*P-value <0.05 in bold. NO_x_ = Nitrogen oxides. MDD = Major Depressive Disorder. Associations show the indirect effect (a*b), the direct effect (c'), and the total effect (c = a*b + c') of air pollution exposure on mental health disorders, with full models including exposure, outcome, assigned sex at birth, family socio-economic status at age 5, neighbourhood deprivation (ages 5-10), urbanicity (ages 5-10), smoking status up to age 18, and the proportion of family members with a psychiatric history. Odds ratios (OR) and 95% confidence intervals (95% CI) represent the increased odds of disorder status between the top quartile of exposure and the bottom three quartiles. All associations are adjusted for non-independence of twin observations with the CLUSTER command. The direct effect represents the direct effect of exposure on MDD odds, independent of overall cognition or crystallised ability at age 12. The indirect effect represents the association between exposure and MDD via overall cognition or crystallised ability.*

# eTable 10: Associations between air pollutant exposures at age 10 (NO_2_, NO_x_, PM_2.5_ and PM_10_) and mental health disorders at age 18 among those who did not move home address between ages 5 and 10

|  | Major depressive disorder | | Generalised anxiety disorder | | ADHD | | Conduct disorder | |
| --- | --- | --- | --- | --- | --- | --- | --- | --- |
| Pollutant | OR | 95% CI | OR | 95% CI | OR | 95% CI | OR | 95% CI |
| NO_2_ | 1.13 | 0.84, 1.52 | 0.88 | 0.54, 1.42 | 0.83 | 0.54, 1.26 | 1.35 | 0.91, 2.01 |
| NO_x_ | 1.17 | 0.89, 1.55 | 0.98 | 0.60, 1.62 | 0.84 | 0.55, 1.27 | 1.33 | 0.94, 1.87 |
| PM_2.5_ | 1.04 | 0.88, 1.24 | 0.96 | 0.72, 1.27 | 0.98 | 0.77, 1.25 | 1.07 | 0.85, 1.34 |
| PM_10_ | 1.02 | 0.88, 1.17 | 1.18 | 0.91, 1.53 | 1.03 | 0.76, 1.38 | 1.07 | 0.89, 1.29 |

*N=1248. NO_2_ = Nitrogen dioxide, NO_x_ = Nitrogen oxides, PM_2.5_= particulate matter size 2.5µm or smaller, PM_10_ = particulate matter size 10µm or smaller. ADHD = Attention-Deficit Hyperactivity Disorder. Associations with full models are presented here, including exposure, outcome, assigned sex at birth, family socio-economic status at age 5, neighbourhood deprivation (ages 5-10), urbanicity (ages 5-10), smoking status up to age 18, and the proportion of family members with a psychiatric history. Odds ratios (OR) and 95% confidence intervals (95% CI) represent the increased odds of disorder status per interquartile range (μg/m^3^) increase in air pollutant levels. All associations are adjusted for non-independence of twin observations with the CLUSTER command.*

# eTable 11: Associations between air pollutant exposures at age 10 (NO_2_, NO_x_, PM_2.5_ and PM_10_) and mental health disorders at age 18 among those who did not move home address between ages 10 and 18

|  | Major depressive disorder | | Generalised anxiety disorder | | ADHD | | Conduct disorder | |
| --- | --- | --- | --- | --- | --- | --- | --- | --- |
| Pollutant | OR | 95% CI | OR | 95% CI | OR | 95% CI | OR | 95% CI |
| NO_2_ | 1.17 | 0.86, 1.59 | 0.82 | 0.47, 1.41 | 1.03 | 0.68, 1.55 | **1.62** | **1.08, 2.42** |
| NO_x_ | 1.22 | 0.91, 1.63 | 0.88 | 0.52, 1.51 | 0.98 | 0.66, 1.45 | **1.53** | **1.11, 2.13** |
| PM_2.5_ | 1.05 | 0.86, 1.28 | 0.87 | 0.64, 1.17 | 1.05 | 0.83, 1.33 | 1.14 | 0.89, 1.45 |
| PM_10_ | 1.04 | 0.89, 1.21 | 1.19 | 0.93, 1.54 | 1.07 | 0.81, 1.42 | 1.12 | 0.93, 1.36 |

*N=1245. NO_2_ = Nitrogen dioxide, NO_x_ = Nitrogen oxide, PM_2.5_= particulate matter size 2.5µm or smaller, PM_10_ = particulate matter size 10µm or smaller. ADHD = Attention-Deficit Hyperactivity Disorder. P-value <0.05 in bold. Associations with full models are presented here, including exposure, outcome, assigned sex at birth, family socio-economic status at age 5, neighbourhood deprivation (ages 5-10), urbanicity (ages 5-10), smoking status up to age 18, and the proportion of family members with a psychiatric history. Odds ratios (OR) and 95% confidence intervals (95% CI) represent the increased odds of disorder status per interquartile range (μg/m^3^) increase in air pollutant levels. All associations are adjusted for non-independence of twin observations with the CLUSTER command.*

# eTable 12: Mediation analysis between NO_x_ exposure at age 10, cognition or crystallised ability at age 12 and MDD at age 18 for participants who did not move between ages 5-10

|  | Direct effect | | Indirect effect | | Total effect | |
| --- | --- | --- | --- | --- | --- | --- |
| Mediator | OR | 95% CI | OR | 95% CI | OR | 95% CI |
| Overall cognition | 1.15 | 0.87, 1.52 | 1.02 | 0.99, 1.05 | 1.17 | 0.88, 1.56 |
| Crystallised ability | 1.16 | 0.88, 1.54 | 1.01 | 0.99, 1.03 | 1.17 | 0.88, 1.56 |

*N=1248. NO_x_ = Nitrogen oxides. MDD = Major Depressive Disorder. Associations show the indirect effect (a*b), the direct effect (c'), and the total effect (c = a*b + c') of air pollution exposure on mental health disorders, with full models including exposure, outcome, assigned sex at birth, family socio-economic status at age 5, neighbourhood deprivation (ages 5-10), urbanicity (ages 5-10), smoking status up to age 18, and the proportion of family members with a psychiatric history. Odds ratios (OR) and 95% confidence intervals (95% CI) represent the increased odds of disorder status per interquartile range (μg/m^3^) increase in air pollutant levels. All associations are adjusted for non-independence of twin observations with the CLUSTER command. The direct effect represents the direct effect of exposure on MDD odds, independent of overall cognition or crystallised ability at age 12. The indirect effect represents the association between exposure and MDD via overall cognition or crystallised ability. The direct effect represents the direct effect of exposure on MDD odds, independent of overall cognition or crystallised ability at age 12. The indirect effect represents the association between exposure and MDD via overall cognition or crystallised ability.*

# eTable 13: Mediation analysis between NO_x_ exposure at age 10, cognition at age 12 and MDD at age 18 for participants who did not move between ages 10-18

|  | Direct effect | | Indirect effect | | Total effect | |
| --- | --- | --- | --- | --- | --- | --- |
| Mediator | OR | 95% CI | OR | 95% CI | OR | 95% CI |
| Overall cognition | 1.22 | 0.91, 1.63 | 1.00 | 0.99, 1.02 | 1.22 | 0.91, 1.63 |
| Crystallised ability | 1.22 | 0.91, 1.64 | 1.00 | 0.99, 1.01 | 1.22 | 0.91, 1.63 |

*N=1245. NO_x_ = Nitrogen oxides. MDD = Major Depressive Disorder. Associations show the indirect effect (a*b), the direct effect (c'), and the total effect (c = a*b + c') of air pollution exposure on mental health disorders, with full models including exposure, outcome, assigned sex at birth, family socio-economic status at age 5, neighbourhood deprivation (ages 5-10), urbanicity (ages 5-10), smoking status up to age 18, and the proportion of family members with a psychiatric history. Odds ratios (OR) and 95% confidence intervals (95% CI) represent the increased odds of disorder status per interquartile range (μg/m^3^) increase in air pollutant levels. All associations are adjusted for non-independence of twin observations with the CLUSTER command. The direct effect represents the direct effect of exposure on MDD odds, independent of overall cognition or crystallised ability at age 12. The indirect effect represents the association between exposure and MDD via overall cognition or crystallised ability.*

# eTable 14: Associations between exposure to NO_x_ at age 10 and MDD at age 18 with E-values and covariates

| **Variables** | **OR** | **95% CI** |
| --- | --- | --- |
|  |  |  |
| **E-value** | 1.48 | 1.08 |
| NO_x_ | 1.25 | 1.01, 1.54 |
| **Assigned sex at birth** |  |  |
| Male | ref |  |
| Female | 1.70 | 1.33, 2.18 |
| **Social class composite** |  |  |
| Low | ref |  |
| Medium | 0.95 | 0.70, 1.28 |
| High | 0.87 | 0.60, 1.25 |
| **Neighbourhood deprivation (average ages 5-10)** |  |  |
| Wealthy achievers | ref |  |
| Urban prosperity | 1.13 | 0.69, 1.86 |
| Comfortably off | 1.07 | 0.72, 1.61 |
| Moderate means | 1.21 | 0.77, 1.91 |
| Hard pressed | 1.17 | 0.74, 1.84 |
| **Urbanicity (average ages 5-10)** |  |  |
| Rural | ref |  |
| Urban city/own | 0.72 | 0.51, 1.02 |
| Major/minor conurbation | 0.66 | 0.42, 1.03 |
| **Proportion of family members with any psychiatric disorder** | 2.91 | 1.85, 4.58 |
| **Ever a daily smoker – up to age 18** |  |  |
| No | ref |  |
| Yes | 2.04 | 1.58, 2.65 |

*NO_x_ = Nitrogen oxides. MDD = Major Depressive Disorder. Associations with full models are presented here, including exposure, outcome, assigned sex at birth, family socio-economic status at age 5, neighbourhood deprivation (ages 5-10), urbanicity (ages 5-10), smoking status up to age 18, and the proportion of family members with a psychiatric history. Odds ratios (OR) and 95% confidence intervals (95% CI) for NO_x_ represent the increased odds of disorder status per interquartile range (μg/m^3^) increase in air pollutant levels. All associations are adjusted for non-independence of twin observations with the CLUSTER command. E values are a statistical reporting of the required effect estimate of any unmeasured confounding that would be required to nullify the association.*


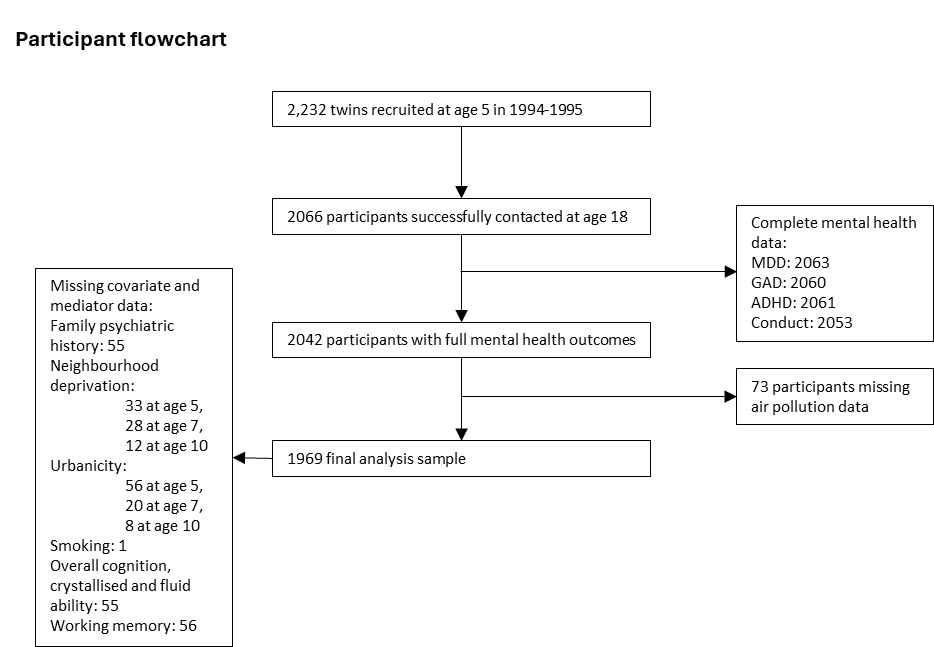


**eFigure 2 Participant flowchart for sample of E-Risk participants included in this study.**  *MDD = Major depressive disorder, GAD = generalised anxiety disorder, ADHD = attention-deficit hyperactivity disorder, Conduct = conduct disorder*


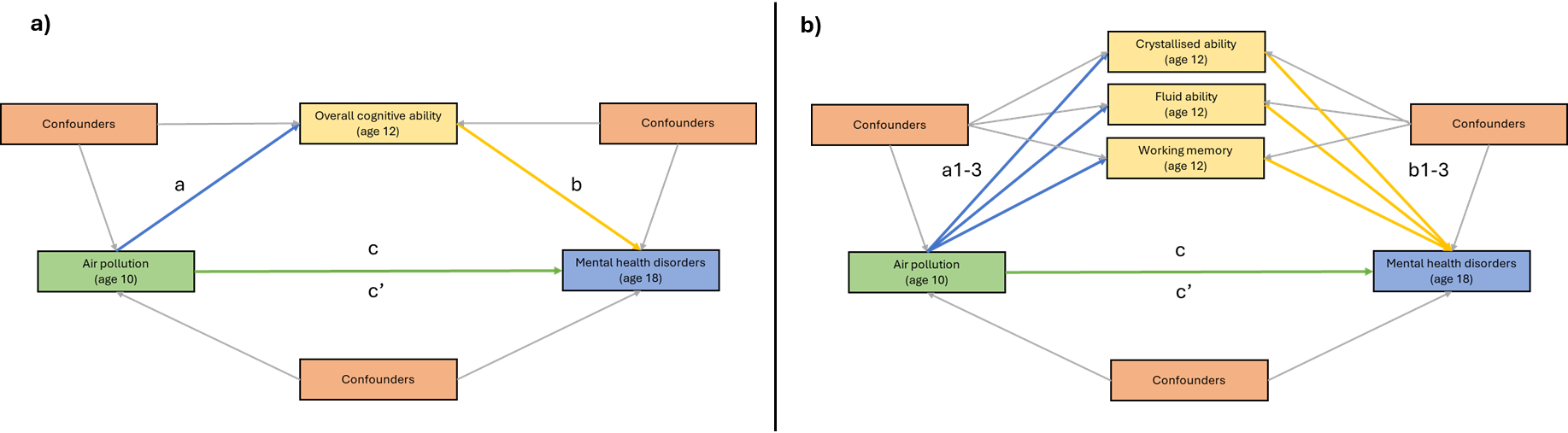


**eFigure 3 Directed Acyclic Graph for exposure, outcome, mediators and confounders proposed in this study.** *a) for overall cognitive ability and b) for cognitive subdomains. Green square = exposure, blue square = outcome, yellow square = mediator, and orange square = confounding variables. Blue and yellow lines represent the indirect effect (a*b), and green lines represent the direct effect (c'), with c representing the total effect (c = a*b + c') of air pollution exposure on mental health disorders. Grey lines represent confounding associations. Confounders are assigned sex at birth, family socio-economic status at age 5, neighbourhood deprivation (ages 5-10), urbanicity (ages 5-10), smoking status up to age 18, and the proportion of family members with a psychiatric history.*
